# Progesterone distribution in the trigeminal system and its role to modulate sensory neurotransmission: influence of sex

**DOI:** 10.1186/s10194-023-01687-x

**Published:** 2023-11-14

**Authors:** Aida Maddahi, Karin Warfvinge, Anja Holm, Jacob C. A. Edvinsson, Philip Victor Reducha, Spyridoula Kazantzi, Kristian A. Haanes, Lars Edvinsson

**Affiliations:** 1 Division of Experimental Vascular Research, Department of Clinical Sciences, Lund University Hospital, Lund, Sweden; 2 Department of Clinical Experimental Research, Glostrup Research Institute, Copenhagen University Hospital, Rigshospitalet Glostrup, Copenhagen, Denmark; 3 Center for RNA Medicine, Department of Clinical Medicine, Aalborg University, Aalborg, Denmark; 4 Department of Biology, Section of Cell Biology and Physiology, University of Copenhagen, Copenhagen, Denmark

**Keywords:** Progesterone, Trigeminal ganglion, Dura matter, Progesterone receptor, CGRP, Migraine

## Abstract

**Background:**

Women are disproportionately affected by migraine, representing up to 75% of all migraine cases. This discrepancy has been proposed to be influenced by differences in hormone levels between the sexes. One such hormone is progesterone. Calcitonin gene-related peptide (CGRP) system is an important factor in migraine pathophysiology and could be influenced by circulating hormones. The purpose of this study was to investigate the distribution of progesterone and its receptor (PR) in the trigeminovascular system, and to examine the role of progesterone to modulate sensory neurotransmission.

**Methods:**

Trigeminal ganglion (TG), hypothalamus, dura mater, and the basilar artery from male and female rats were carefully dissected. Expression of progesterone and PR proteins, and mRNA levels from TG and hypothalamus were analyzed by immunohistochemistry and real-time quantitative PCR. CGRP release from TG and dura mater were measured using an enzyme-linked immunosorbent assay. In addition, the vasomotor effect of progesterone on male and female basilar artery segments was investigated with myography.

**Results:**

Progesterone and progesterone receptor -A (PR-A) immunoreactivity were found in TG. Progesterone was located predominantly in cell membranes and in Aδ-fibers, and PR-A was found in neuronal cytoplasm and nucleus, and in satellite glial cells. The number of positive progesterone immunoreactive cells in the TG was higher in female compared to male rats. The PR mRNA was expressed in both hypothalamus and TG; however, the PR expression level was significantly higher in the hypothalamus. Progesterone did not induce a significant change neither in basal level nor upon stimulated release of CGRP from dura mater or TG in male or female rats when compared to the vehicle control. However, pre-treated with 10 µM progesterone weakly enhanced capsaicin induced CGRP release observed in the dura mater of male rats. Similarly, in male basilar arteries, progesterone significantly amplified the dilation in response to capsaicin.

**Conclusions:**

In conclusion, these results highlight the potential for progesterone to modulate sensory neurotransmission and vascular responses in a complex manner, with effects varying by sex, tissue type, and the nature of the stimulus. Further investigations are needed to elucidate the underlying mechanisms and physiological implications of these findings.

**Graphical Abstract:**

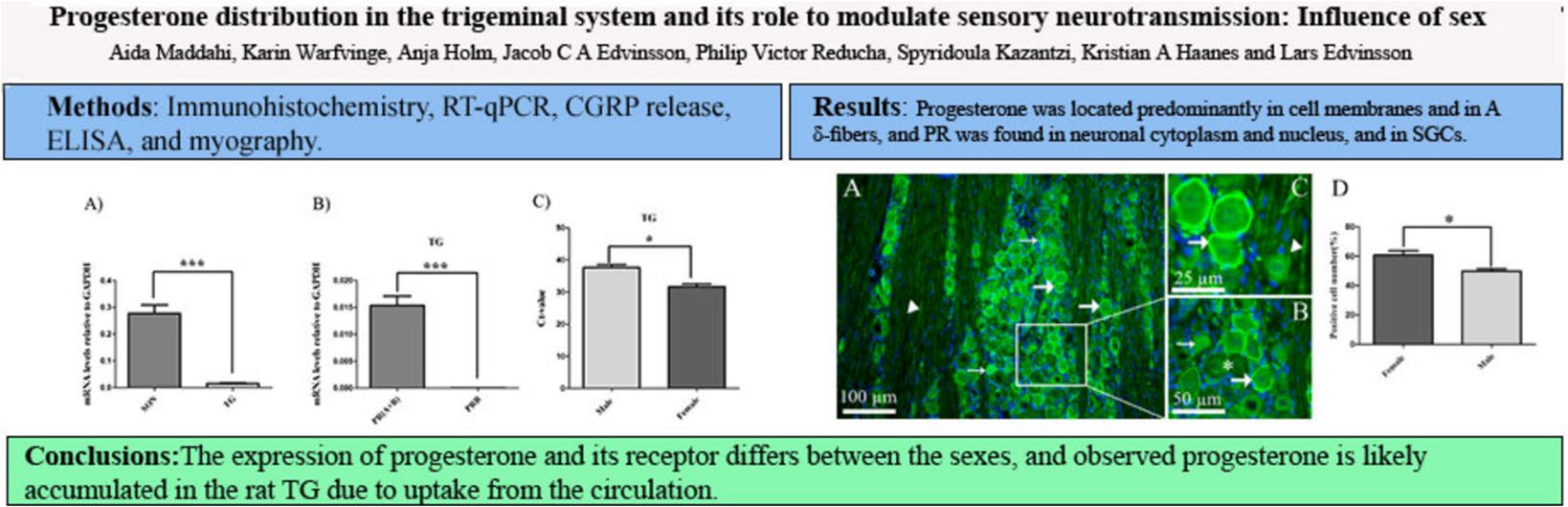

**Supplementary Information:**

The online version contains supplementary material available at 10.1186/s10194-023-01687-x.

## Introduction

Migraine is a chronic, painful, and complex neurological disorder which affects up to 15% of the general population worldwide. Migraine occurs 2–3 times more frequently in females than in males, and often the attacks are associated with onset of menstruation [1, 2]. The sex hormones estrogen, progesterone, and oxytocin show dynamic alterations in plasma with a characteristic drop in circulating levels in close relation to the onset of the menstruation [3, 4].

The beginning of sex hormone secretion and ending with loss of sex hormone sensitivity could frame the migraine process that is driven by sex hormones [5].

Estrogen and progesterone are two ovarian hormones released in different levels depending on time in the menstrual cycle, and they are together with their receptors richly expressed in the cortical and subcortical regions of the brain [6–8].

Migraine attacks vary over a woman’s lifespan, corresponding to altered hormonal states during the menstrual cycle, pregnancy, postpartum and menopause. For example, women with migraine generally experience reduced migraine symptoms by the third trimester. However, migraine can also start during pregnancy, and can worsen for women in the first trimester [9].

Progesterone is a neurosteroid hormone well known for its role in the menstrual cycle. It is produced by the ovaries and placenta in females and also by the adrenal glands and the brain of both sexes [10]. Progesterone can be synthesized locally in the nervous system by glial cells and neurons [11, 12]. The effects of estrogen and progesterone are primarily mediated through binding to intracellular receptors; estrogen receptor alpha (Erα), estrogen receptor beta (Erβ), and G-protein coupled estrogen receptor (GPER), as well as the progesterone receptor (PR) [13–15]. These receptors are members of the nuclear receptor family, which regulate expression of a wide variety of genes at the transcriptional level [6, 16, 17]. The PR mediates physiological effects of progesterone, which plays a central role in reproductive events associated with the establishment and maintenance of pregnancy [18]. PR exists in two isoforms: a smaller isoform, a 94 kDa protein (form A) and a bigger isoform a 120 kDa protein (form B), due to the use of alternative translation initiation sites. PR-B is the transcriptionally active form and is also involved in extra nuclear signaling activation. PR-A is identical to PR-B except that PR-A lacks 164 amino acids at the N-terminus. This deletion exposes a 140 amino acid inhibitory domain (ID) which acts as a repressor of steroid hormone transcriptional activity [18, 19]. Interestingly, pre-clinical research has reported that progesterone plays an important role in the development of neurons in the brain and in its protection from damage and recovery after injury [11, 20, 21]. In addition, progesterone has been found to play a role in promoting the development and repair of the myelin sheath that protects nerve fibers (and neurogenesis), being a way for the hormone to protect and repair the brain [11, 22]. Moreover, a recent study reported the role of progesterone receptors in regulating pain sensitivity and migraine susceptibility in females [23].

While estrogen and progesterone originate mainly in the ovaries another sex hormone (oxytocin) is produced in the magnocellular clusters in the hypothalamus, the paraventricular nucleus (PVN), and the supraoptic nucleus (SON). This hormone distributes to the pituitary gland and the circulation as well as nerve fibers to numerous regions in the brain and brainstem, in particular to the trigeminal nucleus caudalis [24]. Since the trigeminovascular system (TVS) lacks blood–brain barrier [25] circulating molecules, e.g., sex hormones, may reach this region and modify activity and expression of neurons and glial cells in the trigeminal ganglion (TG). Calcitonin gene-related peptide (CGRP) is a sensory neuropeptide consistently shown to be released in excess from TVS following an acute migraine attack [26]. Interestingly, oxytocin, estrogen, and their receptors, are expressed in TG neurons, and are hypothesized to modify expression of CGRP, and its receptors, which is considered to have a key role in migraine pathophysiology [6].

The present study aims to examine the expression and putative function of progesterone and its receptor in the TVS using mRNA, protein expression and functional methods. In addition, we aim to examine co-localization of progesterone and its receptor with CGRP and its receptor. Further, to examine if progesterone may modify the release of CGRP or have direct vascular effects to unravel possible interaction of the sex hormone with aspects of migraine pathophysiology.

## Experimental procedures

### Animals

A total of 60 rats were used in this study (the number of animals in each experiment can be found in Supplementary Table 1). All animal procedures in this study followed the guidelines of the European Communities Council (86/609/ECC) and were approved by the Regional Ethical Committee on Animal Research, Malmö/Lund, Sweden (M17-15) and/or the Danish Animal Experimentation Inspectorate. The animals were housed in Euro standard cages (Type VI with 123-Lid) in groups of 3 animals. Rats were given food and water ad libitum, lived under constant temperature (+ 22 °C) and humidity (55%) and were sustained at a 12/12-h light–dark cycle.

### Tissue preparation

Totally 26 Wistar rats weighing approximately 250–300 g were divided in two groups (15 males and 9 females) and were used for immunohistochemistry and RT-qPCR in this study All animals were anaesthetized using CO_2_ and decapitated. TGs were carefully dissected and were either fixed in 4% paraformaldehyde (PF) in phosphate buffer saline (PBS, Sigma Aldrich, pH 7.2) for 3–4 h at room temperature or fresh frozen in liquid nitrogen. For immunohistochemistry the TGs were cryo-protected with Sörensen’s phosphate buffer (pH 7.2), gradient containing 10% and 25% sucrose overnight. Then, they were embedded in an egg albumin-based protein medium and sectioned at a thickness of 10 µm using a cryostat (Microm Cryo Star HM 560). Finally, the sections were collected on microscope slides (Superfrost™, Merck Chemicals and Life Science, Sweden) and stored at -20 ºC until use.

### Immunohistochemistry

Sections from both male and female rats were washed and permeabilized in PBS containing 0.3% Triton X-100 (PBST) for 15 min. Thereafter, the tissues were blocked for non-specific binding of antibodies for 1 h at room temperature in blocking solution containing PBST, 1% bovine serum albumin (BSA), and 5% normal serum. The sections were incubated over night at 4 °C in moisturized chambers with primary antibodies (for details, see Table 1). The next day, the sections were washed in PBST for 3 × 15 min and incubated with secondary antibodies (for details, see Table 2) for 1 h at room temperature and kept in the dark to minimize loss of fluorescence. All antibodies were diluted in PBST containing 1% BSA. The sections were subsequently washed with PBST for 3 × 15 min and mounted with anti-fading Vectashield mounting medium containing 4', 6-diamidino-2-phenylindole (DAPI) (Vector Laboratories, Burlingame CA, USA). The described procedure was performed in triplicate for each animal to ensure reproducibility. Further, negative controls were included by omitting the primary antibody to evaluate auto-fluorescence and non-specific secondary antibody binding. Immunoreactivity was visualized using an epifluorescence microscope (Nikon 80i; Tokyo, Japan) at the appropriate wavelengths and photographed with an attached Nikon DS-2Mv camera. Images were processed using Adobe Photoshop CS3 (v0.0 Adobe Systems, Mountain View, CA).

**Table 1:** Primary antibodies used for immunohistochemistry

Name and product code	Dilution	Host	Detects	Supplier
Progesterone BP169	1:50	Rabbit	Rat progesterone	ORIGENE, Rockville, USA
Progesterone (NB100-65167)	1:100	Rabbit	Rat progesterone	Novus Biologicals, USA
Progesterone receptor (MA5-12,658)	1:50	Mouse	PR from a human endometrial carcinoma, detection of PR-A	Thermo Fisher Scientific, USA
Progesterone receptor,ab101688	1:100	Rabbit	Human progesterone receptor aa 400–500, detection of PR-A	Abcam, Cambridge, UK
Progesterone receptor (MA5-14,505)	1:100	Rabbit	Human progesterone receptor 412–526 aa, detection of PR-A	Thermo Fisher Scientific,USA
Progesterone receptor (MA1-411)	1:50	Mouse	Progesterone receptor, detection of PR-B	Thermo Fisher Scientific,USA
Progesterone receptor,SC-810	1:50	Mouse	Human progesterone receptor, detection of PR-A and PR-B	Santa Cruz Biotechnology, Germany
CGRP(ab81887)	1:100	Mouse	Rat α-CGRP	Abcam, Cambridge, UK
RAMP1(844)	1:100	Goat	C-terminal of rat RAMP1	Merck & Co., Inc., USA
CLR 3155	1:500	Rabbit	C-terminal of rat CLR	Merck & Co., Inc., USA
GI Syn (sc-74430)	1:50	Mouse	Raised against amino acids 1–373 representing full length GI Syn of human origin	Santa Cruz, Biotechnology, Germany

**Table 2:** Secondary antibodies used for immunohistochemistry

Name	Dilution	Against	Supplier
Alexa 488	1:100	Goat	Thermo Scientific, IL, USA
Alexa 594	1:200	Mouse	Thermo Scientific, IL, USA
FITC	1:100	Mouse	Jackson Immunoresearch, West Grove, PA, USA
Alexa594	1:200	Rabbit	Thermo Scientific, IL, USA
FITC	1:100	Rabbit	Jackson Immunoresearch, West Grove, PA, USA

Five different antibodies were selected to detect expression of PR, and two different antibodies were chosen to detect expression of progesterone in TG (Table 1). One antibody from ThermoFisher with catalog number MA5-12,658 was chosen to represent the visualization of the results of PR as well as antibody from Novus Biologicals (Cat.No. NB100-65167) was chosen to represent the visualization the results of progesterone in the figures.

### Double immunohistochemistry

Double immunohistochemistry was performed using antibodies against progesterone and PR in combination with CGRP and/or its receptor components calcitonin receptor-like receptor (CLR) and receptor activity modifying protein 1 (RAMP1), or astrocyte and satellite glial cell marker glutamine synthetase (GI Syn). The antibodies were applied and mixed as a cocktail. All procedures were the same as described above.

### Cell counting

Cell counting was performed to semi-quantify the expression of progesterone in TG. Three slides with three sections on each were used for measurements. Counting of cells which had visible nuclei was performed in the thickest part of pooled ophthalmic-maxillary and mandibular areas. Due to the risk of artefactual fluorescence, counting of neurons close to the TG surface was not performed. Images were taken of the screen (0.75 mm^2^) at 10 × magnification. A NIS-elements BR image analysis program (Nikon) was used to calculate the number of cells and to measure the fluorescence intensity in each area. All cells in this area, including negative and immunoreactive cells, were counted. The mean percentage of positive neurons in 3 slides/rat, from all rats (*n* = 6) was used for analysis. The intensity measurements were used to verify that immunoreactive cells were correctly distinguished from negative cells.

### RNA isolation and RT-qPCR

We performed two different experiments with real-time quantitative PCR (RT-qPCR). First, rat hypothalamus and TGs from eight male rats were carefully dissected, and immediately frozen in liquid nitrogen for RNA extraction. All RNA extraction was performed using RNeasy® Plus Mini kit (Qiagen, Hilden, Germany) in accordance with the manufacturer’s protocol. Total RNA concentration was determined using a GeneQuant Pro spectrophotometer (Amersham Pharmacia Biotech, Uppsala, Sweden). A ratio of sample absorbance at 260 /280 nm in the range of 1.8 to 2 was considered acceptable. First-strand cDNA was prepared from 1 µg of total RNA (from hypothalamus and TG) in a 20 µL reverse transcript reaction using Superscript® III First-Strand Synthesis Super Mix (Invitrogen, Carlsbad, CA, USA). A reverse transcription negative control for each sample to detect the genomic DNA was performed simultaneously and underwent the same procedures but without Superscript III Reverse Transcriptase (RT enzyme). The cDNA obtained was diluted to a total volume of 80 µL and stored at -20 °C. The primer sequences were specific for the genes of interest and were designed using Primer Express 3.0 software (PE Applied Biosystems, Foster city, CA, USA) and synthesized by TAG Copenhagen A/S (Copenhagen, Denmark). The housekeeping gene glyceraldehyde-3-phosphate dehydrogenase (GAPDH) was used as a reference gene and the gene expressions were normalized versus that. Primers had the following sequences:

PR (A + B) *(*forward; *5`- CTGCTGGATGAGCCTGATGGTG-3*`, revers; *5`- CACCATCCCTGCCAGGATCTTG-3`);* PR-B (forward*; 5`-CAGACCAACCTGCAACCAGAA -3`,* revers*; 5`-AGTCCTCACCAAAACCCTGGG-3*`*);* GAPDH *(*forward*; 5`-CTGCACCACCAACTGCTTAGG -3`,* revers*; 5´-TCAGCTCTGGGATGACCTTGC- 3`).*

The RT-qPCR was performed in 20 µL reaction consisting of 2 µL diluted cDNA, 0.6 µM of each primer, 10 µL Fast SYBR™ Green Master Mix (Applied Biosystems, CA, USA), and 7 µL RNase free water in a Step One Plus Real Time PCR System (Applied Biosystems, CA, USA) with the following thermal profile: Holding stage at + 50 °C for 2 min, + 95 °C for 10 min, followed by 40 PCR cycles at + 95 °C for 15 s and + 60 °C for 1 min. Each sample was examined in duplicate, and a blank control (without template) was used in all experiments. After amplification a melting curve analysis was performed to confirm specificity of primers annealing and to verify that each primer pair generated only one PCR product of expected size.

The second experiment was designed to analyze the expression level of PR in three male and three female rats. Total RNA was extracted from the TG using spin columns (NucleoSpin miRNA, Mini kit for total RNA, MACHERY-NAGEL) in combination with QIAzol (Qiagen, Germany) and chloroform (Sigma Aldrich, Denmark). The samples were homogenised using QIAzol lysis buffer (Qiagen, Germany) and 1.4 mm ceramic beads (Lysing Matrix D, MP Biomedicals, USA) for 40 s at max speed using a FastPrep-24TM 5G instrument (MP Biomedicals, USA). The RNA concentration was measured using a Nanodrop 2000c (ThermoFisher, USA) at 260 nm. 1 µg RNA was reverse transcribed using the iScript cDNA Synthesis Kit (Biorad, USA) according to the manufacturer´s protocol. RT-qPCR was performed using 20 × pre-designed TaqMan rat specific gene expression assay (IDT, USA), Prime Time Gene expression Master mix, and analysed using the Quant-Studio 6 Pro Real-Time PCR system (Applied Biosystems, USA). The thermal cycling condition included an initial denaturation step at + 50 °C for 2 min and + 95 °C for 10 min followed by 40 PCR cycles at + 95 °C C for 15 s and + 60 °C for 1 min. Pre-designed TaqMan gene expression assays used in this study detects all transcript variants and were purchased from IDT: PGR: Rn.PT.58.10589420 and ACTB: Rn.PT.39a.22214838.g.

### CGRP Release

Additional five male and five female rats were subjected to anaesthesia using CO_2_ before decapitation. TGs were carefully dissected and immersed in a 10 mL solution of synthetic interstitial fluid (SIF), comprising 108 mM NaCl, 3.5 mM KCl, 3.5 mM MgSO_4_, 26 mM NaHCO_3_, NaH_2_PO_4_, 1.5 mM CaCl_2_, 9.6 mM NaGluconate, 5.6 mM glucose, and 7.4 mM sucrose, maintained at a temperature of + 37 °C for a period of 30 min. Subsequently, these TGs were transferred into Eppendorf tubes positioned on a + 37 °C heating block and subjected to a rinsing process of four cycles, each lasting for 10 min with 300 µL of SIF, similar as previously published [24, 27].

To probe the release from the dura mater, the hemispheres of the brain were carefully excised from the cranium while keeping the cranial dura attached to the skull. The skull halves were then placed into a container filled with 250 mL of SIF and subjected to a dual rinse cycle, each lasting for 15 min. Both skull halves were maintained in a humid chamber placed above a water bath to ensure a constant temperature of + 37 °C. The skulls were then washed four times with 300 µL SIF, with each wash lasting 10 min.

After a 15-min incubation period with 300 µL SIF, samples of 200 µL for measuring basal CGRP release were obtained from all tissues, combined with 50 µL enzyme immunoassay buffer, and preserved at -80 °C for future analysis within two weeks from the time of the experiment. Prior studies have validated the absence of significant variations between basal CGRP release from left and right tissue sides, enabling the use of one side for progesterone testing, while the other served as a vehicle control. This paired design was leveraged to minimize experimental and biological variations within this assay [28, 29].

The CGRP release from the TG samples and the cranium halves were initially assessed under basal conditions, followed by an evaluation under the influence of progesterone (Cat. No. 2835, Biotechne, Ireland) dissolved in DMSO (Sigma-Aldrich, Germany) after a 15-min incubation to test for potential progesterone-induced release. Subsequently, in the presence of the same concentrations of progesterone or vehicle (DMSO), 100 nM capsaicin (211,275, Sigma-Aldrich, Germany) was utilized to stimulate the release of CGRP. Prior experiments have determined that 10-min incubation duration is sufficient for significant and reproducible CGRP release above baseline levels [29]. The workflow can be found in Supplementary Fig. 1. CGRP release was quantified using commercial Enzyme Immunoassay (EIA) kits (SPIbio, Paris, France). The CGRP concentration was calculated based on the standard curve, and the data was normalized against its contralateral control. The CGRP EIA reagent contains antibodies directed against human-CGRP but demonstrating equal reactivity with rat and mouse CGRP. The protocol adhered to the manufacturer's guidelines. The optical density was measured at 410 nm using a microplate photometer (Tecan, Infinite M200, software SW Magellan v.6.3, Mannedorf, Switzerland).

### Ex vivo vasomotor responses of basilar arteries: myography

Rats (*n* = 12 of either sex) were sedated with a mixture of O_2_/CO_2_ (30/70%) and euthanized by decapitation. Following euthanizing, the basilar artery was immediately extracted and submerged in a chilled, oxygenated physiological solution of Na-Krebs buffer (consisting of: NaCl 119 mM, NaHCO_3_ 15 mM, KCl 4.6 mM, MgCl_2_ 1.2 mM, NaH_2_PO_4_ 1.2 mM, CaCl_2_ 1.5 mM, and glucose 5.5 mM). Ring segments of approximately 2 mm in length were prepared for myography studies [30, 31] and mounted on 40 µm wires. These arterial segments were allowed a 30-min equilibration period before being expanded to their optimal lumen diameter L1 = 0.9 × L100, where L100 refers to the diameter of the vessel under a passive transmural pressure of 100 mmHg (13.3 kPa). Initial evaluation of the contractile capacity of each arterial segment was conducted by exposing them to 60 mM K^+^. Following this, a concentration–response curve for contractile (from baseline) or dilation (from a preconstruction with 100 nM U46619 (Cayman #16,450, BioNordika, Denmark) was developed via the cumulative addition of progesterone. U46619 is a potent thromboxane A2 analog that primarily functions as a vasoconstrictor, inducing smooth muscle contraction in blood vessels. Furthermore, the dilation response to capsaicin in the presence of 10 µM progesterone/vehicle (DMSO) was tested.

## Calculation and statistical analyses

RT-qPCR data were analyzed with comparative cycle threshold (C) method [32]. Fold change and standard deviations were calculated using the ∆∆CT method.

All statistical analyses were performed using Graph Pad Prism 9. Statistical significance for RT-qPCR were determined using Mann–Whitney, Student’s t-test and for CGRP release a paired two-tailed Student's t-test and for the myograph data utilizing two-way ANOVA with Sidak post-test. Data were expressed as mean ± standard error of the mean (SEM), *n* refers to the number of rats, *P < 0.05, **P < 0.01 and ***P < 0.001 were considered significant.

## Results

### Immunohistochemistry

Indirect immunohistochemistry was used for detection and localization of progesterone and PR-A protein expression in TGs of male and female rats.

#### Distribution of progesterone and progesterone receptor in the trigeminal ganglion

**Fig. 1:**
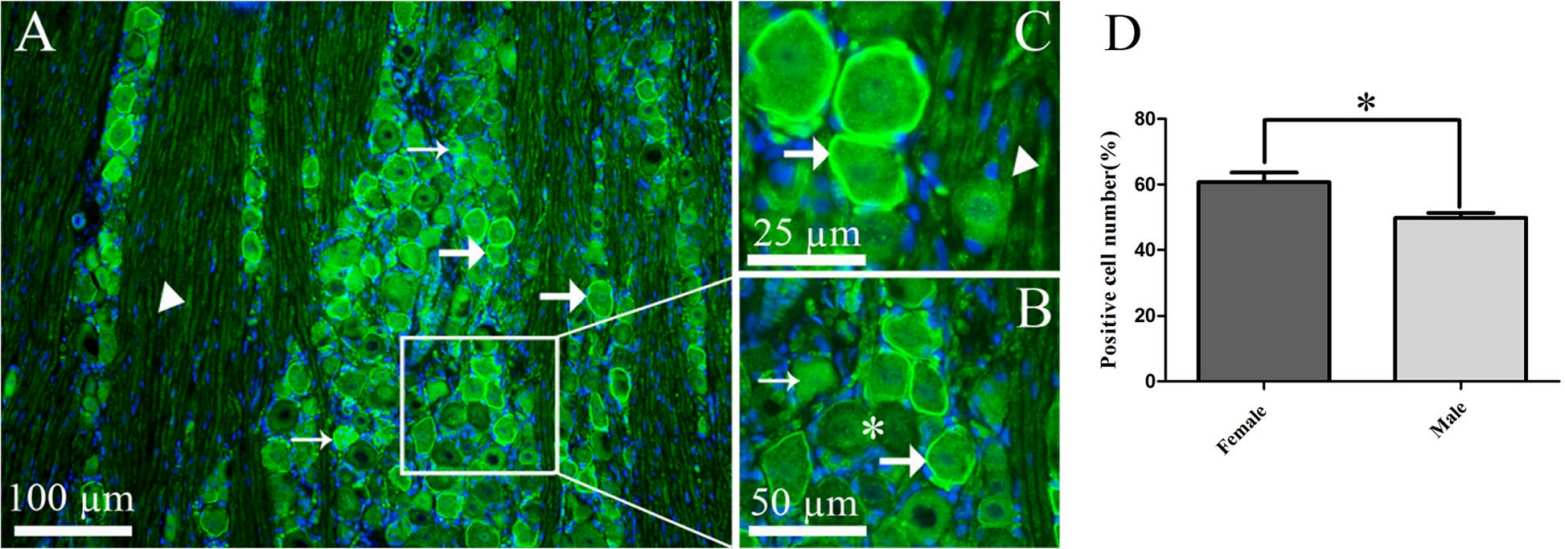
Progesterone immunoreactivity in TG. **A-C** Progesterone was expressed in the cell surface of TG neurons (thick arrows) and had a weak expression in Aδ-fibers (arrowheads). In addition, progesterone immunoreactivity was seen in the cytoplasm of a few small to medium sized neurons (thin arrows, A and B). Negative cells are marked with asterisk in Fig. 1B. **B** and **C** showing higher magnification of image A. Blue colors represent nucleus staining with DAPI. **D** The bar graphs show progesterone immunoreactive cells in females (60.6% ± 7) and males (49% ± 4) of the total number of neurons. This was significantly higher in female rats. Data are presented as the mean ± SEM, *n* = 6, and *P < 0.05

Progesterone immunoreactivity was expressed in/or close to the cell membrane of large to medium-sized neurons and in thick nerve fibers (presumably Aδ-fibers) (Fig. 1A-C). In addition, diffuse progesterone immunoreactivity was seen in the cytoplasm of some neurons (Fig. 1A and B). Further, the number of progesterone expressing neurons was calculated according to the method described above. Neurons that showed visible cell membrane staining like a ring around the cells together with neurons with obvious cytoplasm intensity were counted as positive neurons. It was found that the number of progesterone positive neurons (of the total number of neurons) was significantly (*P* < 0.05) higher in female rats (60.6% ± 7) compared to male rats (49% ± 4) (Fig. 1D).

Five different antibodies were selected to detect the expression of PR-A, PR-B, or PR (A + B) to confirm and ensure the results (see Table 1). All antibodies, which detect the PR-A isoform of PR, displayed similar results. To the contrary, there was no staining with antibodies which detect the PR-B isoform.

**Fig. 2:**
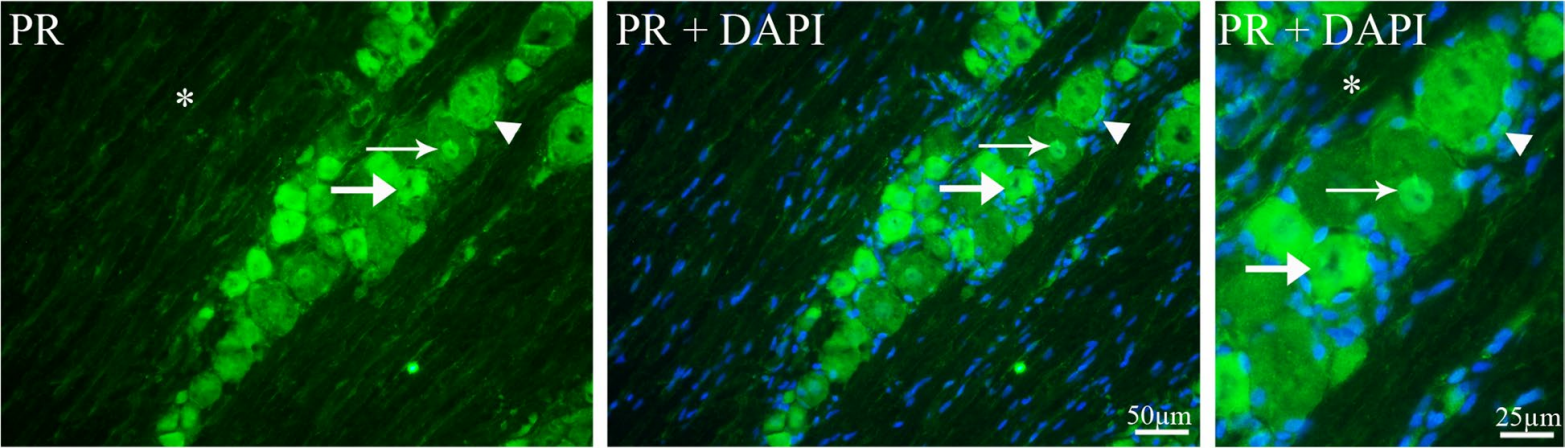
Progesterone receptor immunoreactivity in TG. PR immunoreactivity was localized in neuronal nucleus (thin arrows) and in cytoplasm (thick arrows). In addition, PR immunoreactivity was seen in the cytoplasm of satellite glial cells (arrowheads). There was observed a very weak expression of PR in the Schwann cells (asterisk). Blue colors represent nucleus staining with DAPI

PR-A immunoreactivity was observed in neuronal nucleus and cytoplasm, and in the cytoplasm of SGCs (Fig. 2). In some neurons PR-A immunoreactivity was located only in the nuclei but in other neurons immunoreactivity was in both nucleus and cytoplasm (Fig. 2). There was no PR immunoreactivity in fibers; however, a very weak immunoreactivity was seen in glial cells (probably Schwann cells). In addition, PR-A positive neurons were counted and approximately 50—60% of all neurons expressed PR-A immunoreactivity (59.6% ± 2.3 in females and 53.2% ± 2 in males).

#### Co-localization of progesterone with CGRP and RAMP1

**Fig. 3:**
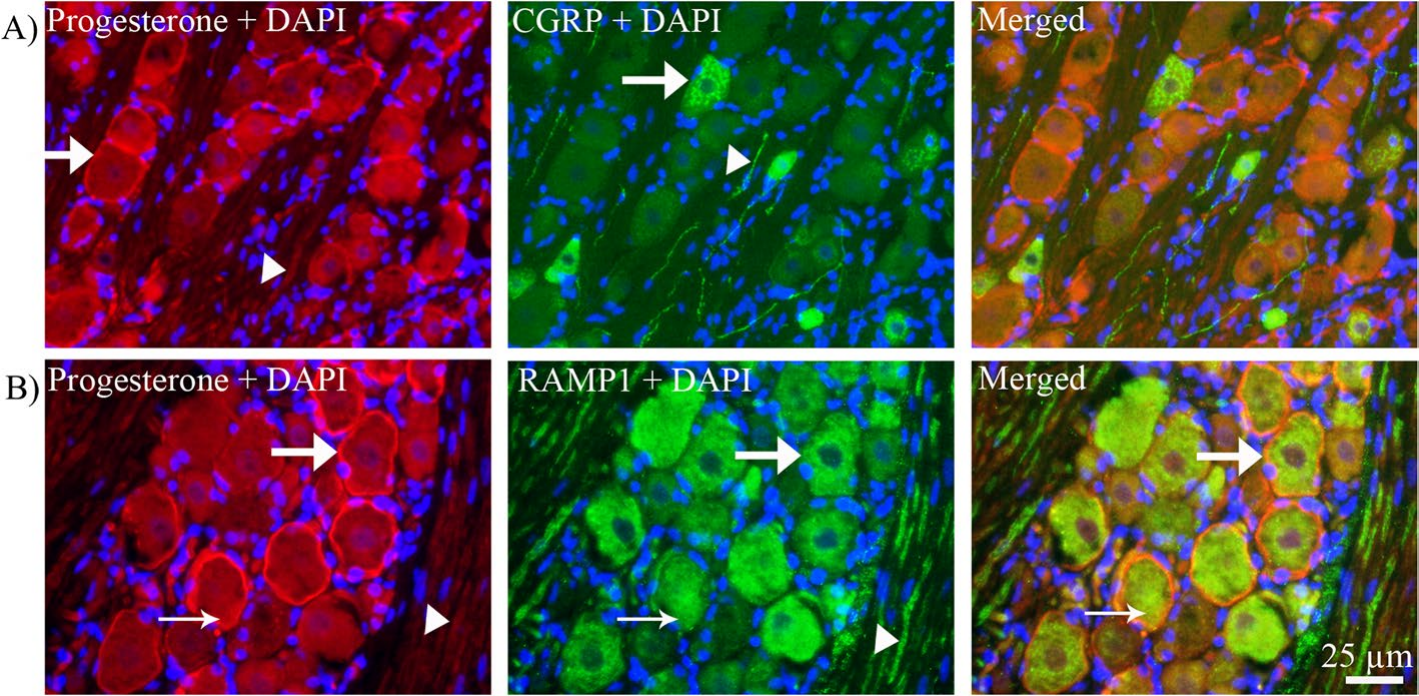
Double immunohistochemistry of progesterone and CGRP or RAMP1. **A** *Progesterone and CGRP*: Progesterone (red) and CGRP (green) immunoreactive neurons (arrows) and in fibers (arrowheads) and their co-localization in the merged picture showed that there was no co-localization between them. **B** *Progesterone and RAMP1*: Double staining of progesterone and RAMP1 showed that in some neurons progesterone (red) and RAMP1 (green) expressed in same type of neurons but in different location (arrow in the merged image). Progesterone was most prominently expressed in the assumed cell membrane with some immunoreactivity within the cytoplasm, while RAMP1 was mainly localized in the cell cytoplasm

Double immunohistochemistry was performed to investigate co-localization of progesterone with CGRP and RAMP1. There was no co-localization between progesterone (expression in cell membrane) and CGRP (expression in neuronal cell cytoplasm) neither in neurons nor in fibers (Fig. 3A).

RAMP1 and progesterone were mostly co-expressed in the same type of neurons although progesterone was expressed in or close to the cell membrane (Fig. 3B) and RAMP1 was expressed in the cytoplasm.

#### Co-localization of progesterone with GI Syn

**Fig. 4:**
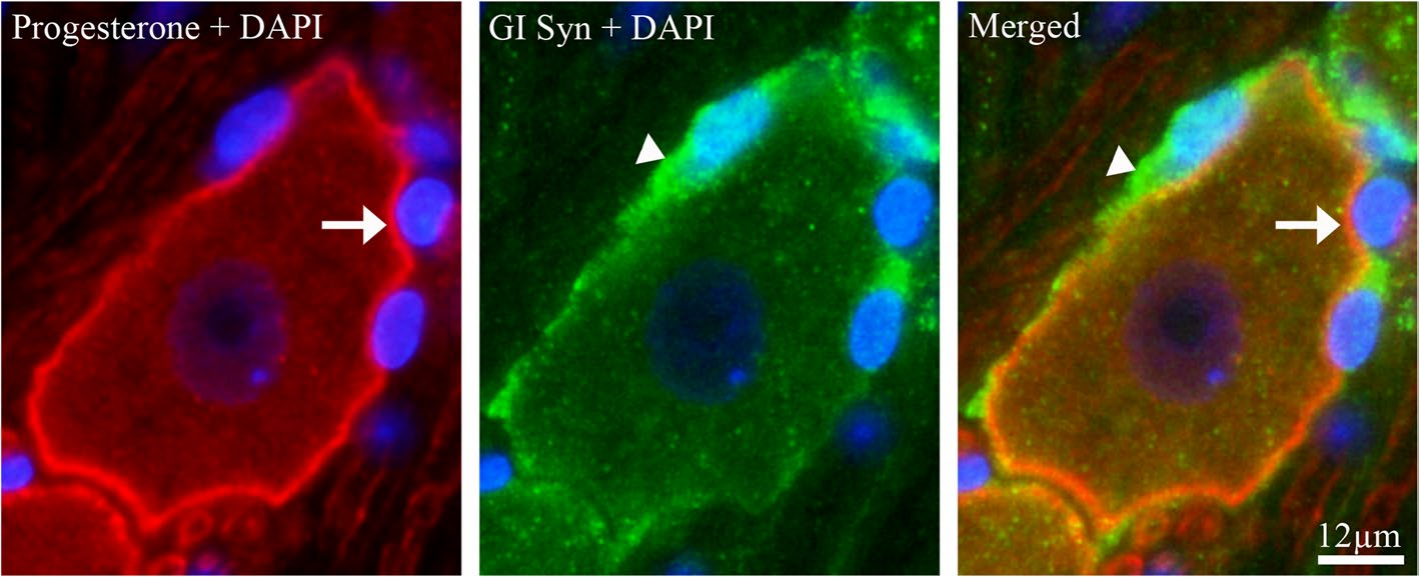
Double immunohistochemistry of progesterone and GI Syn in TG. Arrows indicates progesterone expression in surface of neurons (red) and arrowheads indicates GI Syn expression in satellite glial cells (green). Results showed that there was no co-localization between progesterone and GI Syn

To investigate if progesterone was expressed in the neuronal cell membrane or in the closely associated satellite glial cells, double immunohistochemistry was performed with progesterone antibodies and an enzyme and marker for astrocytes and glial cells (GI Syn). We found no co-localization between progesterone and GI Syn (Fig. 4).

#### Co-localization of progesterone receptor with CGRP, RAMP1 and CLR

**Fig. 5:**
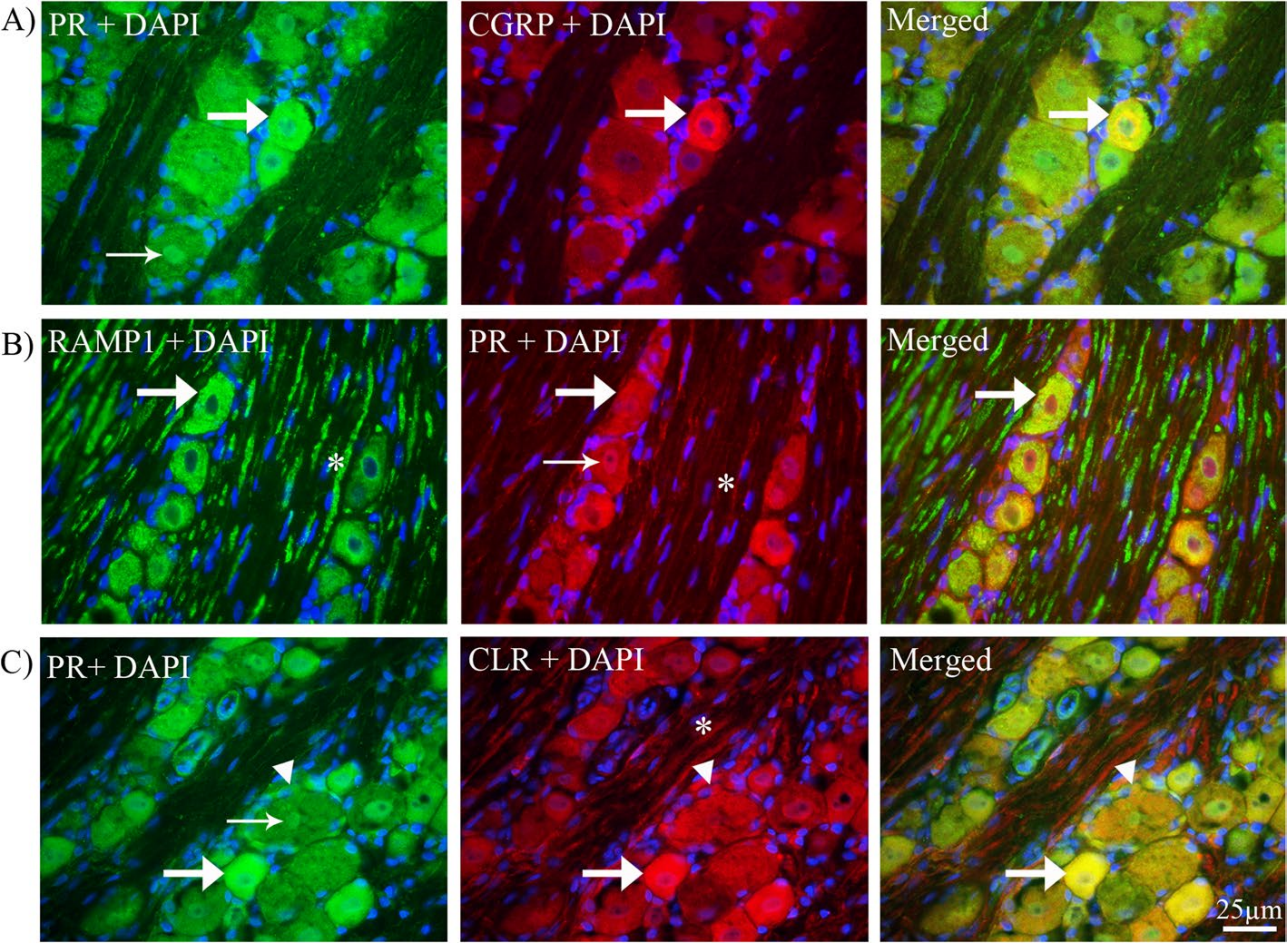
Double immunohistochemistry of PR with CGRP, RAMP1 or CLR in TG. **A** *PR and CGRP*: PR and CGRP immunoreactive cells in neurons (thick arrows) and their co-localization in the merged image (thick arrow) showed that PR co-localized with CGRP in the cytoplasm of small to medium sized neurons. **B** *PR and RAMP1:* RAMP1 was expressed in the cytoplasm of neurons (thick arrow) and in Aδ-fibers (asterisk). PR was expressed in the cytoplasm of neurons (thick arrow), in the neuronal nucleus (thin arrow). In addition, a weak expression was seen in Schwan cells (asterisk). We observed that PR co-localized with RAMP1 in the cytoplasm of medium to large sized neurons (thick arrow in merged image). **C** *PR and CLR*: PR co-localized with CLR in the cytoplasm of neurons (thick arrows) and in the satellite glial cells (arrowheads) but not in the fibers. Examples of RAMP1 and CLR immunoreactive fibers are marked with an asterisk

Double immunohistochemistry showed that PR-A and CGRP were co-expressed in the neuronal cytoplasm of small and medium-sized neurons (Fig. 5A).

Next, evaluation of PR co-localization with RAMP1 was performed. The results showed that PR and RAMP1 were co-expressed in the cytoplasm of some medium- sized neurons (Fig. 5B). In addition, CLR immunoreactivity was observed in neuronal cytoplasm, in the cytoplasm of satellite glial cells and in Aδ-fibers (Fig. 5C). Further, it was found that PR-A co-localized with CLR in the cytoplasm of both TG neurons and satellite glial cells (SGCs) (Fig. 5C). However, we could not find any co-localization between PR-A and CLR in fibers.

### The mRNA expression of progesterone receptor in hypothalamus and TG

To determine whether the PR genes are expressed in hypothalamus and TG, gene expression analysis was performed. In each RT-qPCR experiment either a no temple control (water control) or a minus RT control was included, and there were no signs of contamination, primer-dimer and/or genomic DNA in those samples.

**Fig. 6:**
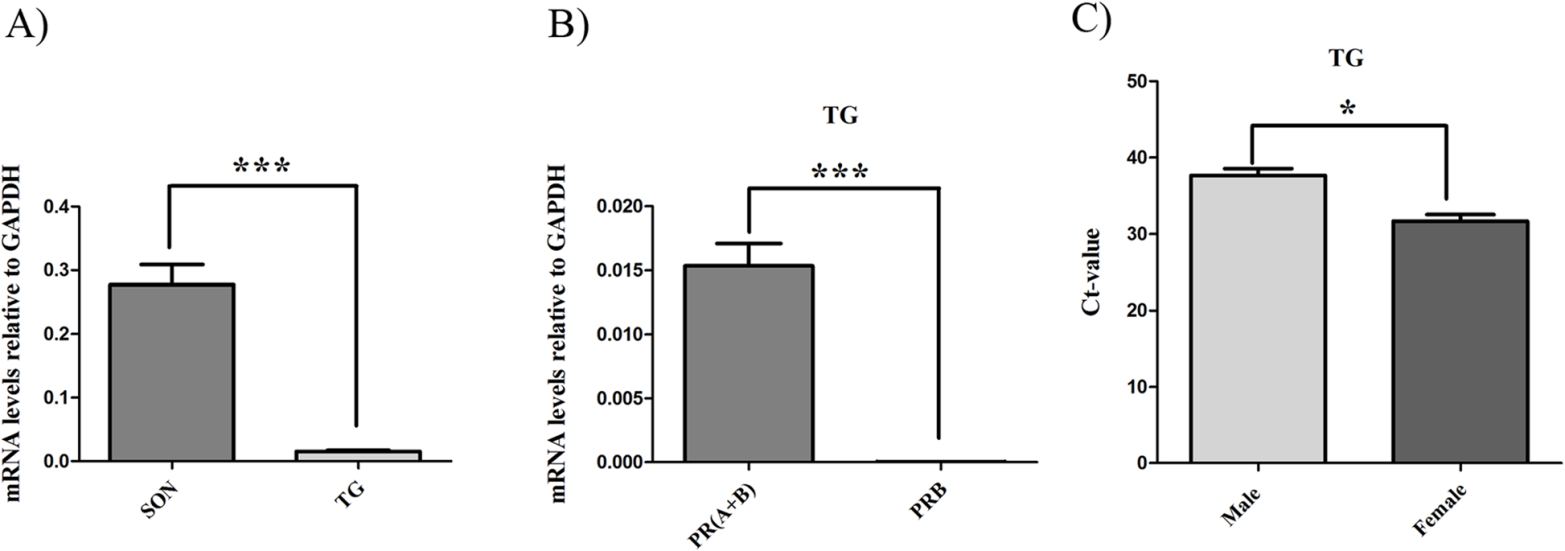
Expression of PR gene in hypothalamus and TG.** A** PR mRNA level in Supraoptic nucleus (SON) in hypothalamus and in TG indicate an abundant PR mRNA expression in hypothalamus. This was significantly higher in comparison to PR mRNA level in TG. **B** PR mRNA levels in TG indicate that the expression of PR (A + B) mRNA was significantly higher than PR-B mRNA. Expressions were normalized to housekeeping gene GAPDH,* n* = 8. **C** The detection level of PR mRNA was significantly higher in female compared to male rats, *n* = 3. Data were obtained by RT-qPCR and are expressed as mean ± SEM, *P < 0.05, **P < 0.01 and ***P < 0.001

The results shown in Fig. 6A demonstrate that there was expression of PR (including A + B) mRNA in supraoptic nucleus cells located in the hypothalamus (Ct: 20.51 ± 0.1) and in TG (Ct: 24.12 ± 0.1). However, the expression of PR mRNA in hypothalamus was significantly higher than in TG. In addition, we investigated the expression of both PR-B and PR (including both PR-A + PR-B) mRNAs in TG and found that the level of PR-B mRNA was low in the TG (Ct: 33.29 ± 0.07) in comparison with the PR mRNA level, which significantly was higher (Ct: 24.1 ± 0.1) (Fig. 6B). This indicates that the expression of PR-A mRNA is more abundant than PR-B mRNA in TG. We also quantified the expressional level of PR (including A + B) in TG from male and female rats and found that female rats had a significantly higher level of PR compared to male rats (Fig. 6C).

### CGRP Release

**Fig. 7:**
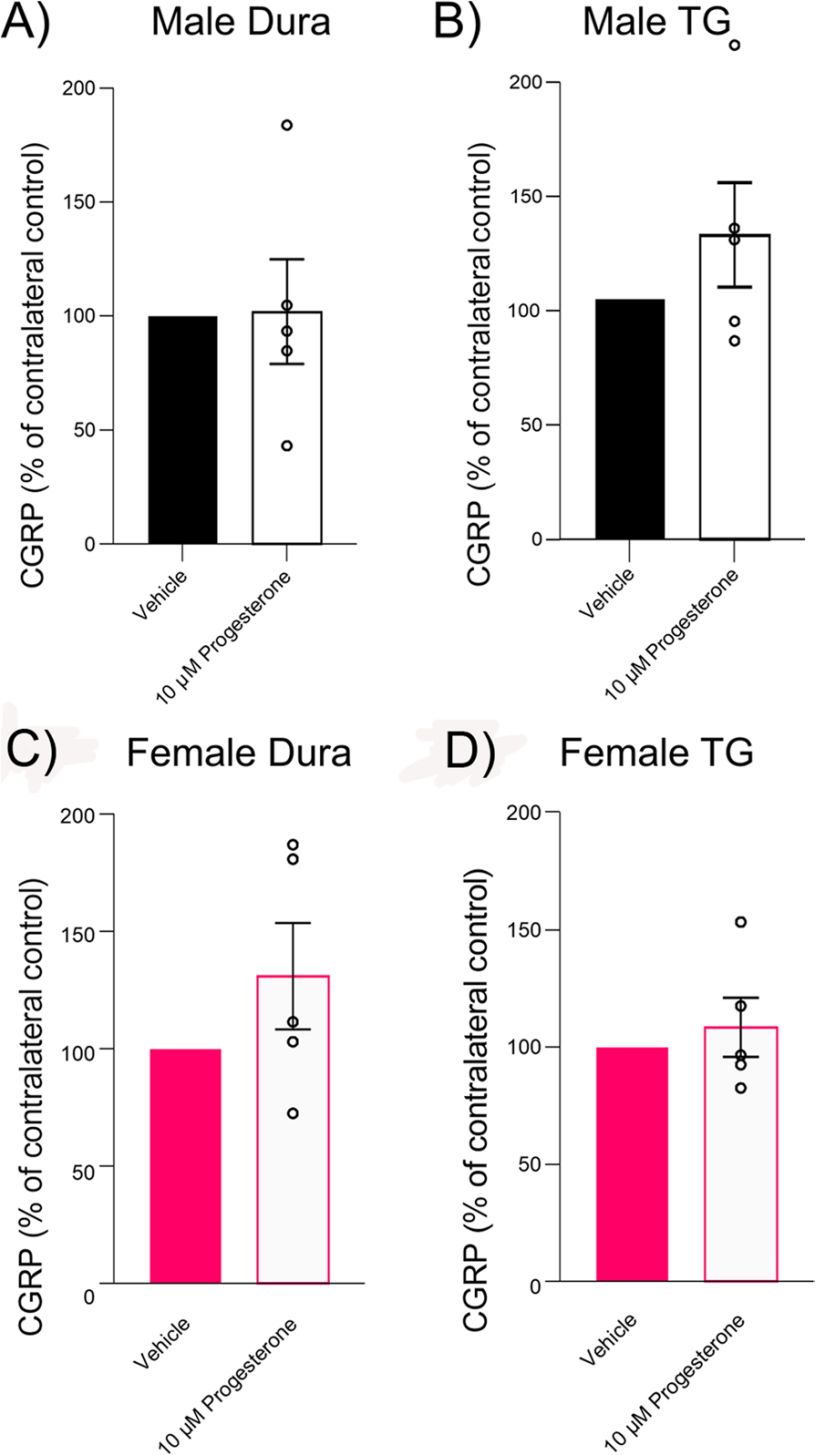
Effect of progesterone on CGRP release from dura mater and TG in male and female rats. Displayed in the four panels are the CGRP release profiles for male dura mater (**A**), male TG (**B**), female dura mater (**C**), and female TG (**D**). In each instance, the tissues were exposed to 10 µM progesterone or vehicle, and the subsequent CGRP release is illustrated. The data, represented as mean ± SEM, (*n* = 5), are expressed as a percentage of the vehicle control, indicating no significant impact of progesterone on CGRP release across all conditions

The effects of progesterone on the release of CGRP were investigated in the dura mater using the hemi-skull approach, and the dissected TG from both male and female rats. The findings were compared with a control group, which consisted of the contralateral hemi-skull or opposite TG exposed to a vehicle (DMSO), and the results are presented as percentages of the vehicle control (Fig. 7). In male rats, the CGRP release from the dura mater (Fig. 7A) and TG tissues (Fig. 7B) after exposure to 10 µM progesterone was 101.9% ± 22.9 and 126.6% ± 21.8 of the vehicle control, respectively. For female rats, the CGRP release from the dura mater (Fig. 7C) following exposure to 10 µM progesterone (in DMSO) was 131.1% ± 22.6 of the vehicle (DMSO) control. The release from the dissected TG **(**Fig. 7D) under the same conditions was 108.5% ± 12.6 of the vehicle control. No significant difference was observed between male and female rat tissues. In summary, the data indicate that progesterone does not induce a significant difference in the release of CGRP from dura mater or TG tissues in male or in female rats when compared to the vehicle control.

**Fig. 8:**
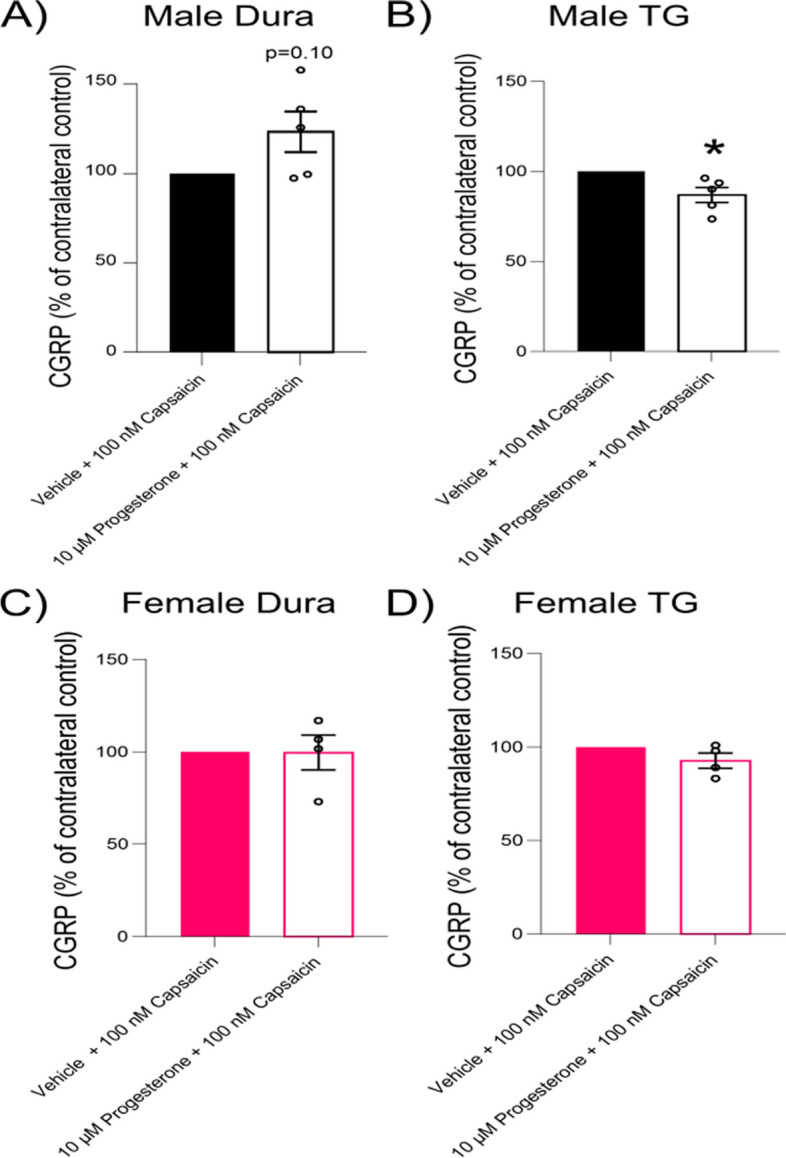
Examination of progesterone's modulation on capsaicin induced CGRP release from rat dura mater and TG. The impact of progesterone on capsaicin induced CGRP release in both male and female rat tissues. The panels illustrate CGRP release following pre-treatment with 10 µM progesterone/vehicle and subsequent addition of 100 nM capsaicin in male dura mater (**A**), male TG (**B**), female dura mater (**C**), and female TG (**D**). In the male dura mater, there is a trend towards increased CGRP release, whereas a significant reduction is observed in the male TG. No substantial changes are noted in female tissues. Data are presented as mean ± SEM (*n* = 5) and are displayed as a percentage of the vehicle control. Statistical significance was determined using Student's t-test, with * = *P* < 0.05 indicating a significant difference

We further explored the potential influence of 10 µM progesterone on capsaicin-induced release of CGRP from dura matter and TG tissues in both male and female rats. For this purpose, 100 nM capsaicin was applied after the introduction of progesterone or the vehicle, and subsequent CGRP release was assessed (for workflow see Supplementary Fig. 1). The results are presented relative to the release seen following treatment with vehicle and capsaicin, as the study was not designed to detect differences between males and females for capsaicin induced release. The raw data in pg/ml can be found in Supplementary Table 2. In the case of female rats, progesterone did not significantly affect the capsaicin induced CGRP release from either the dura mater (Fig. 8A) or the TG tissues (Fig. 8B), with recorded releases of 99.65% ± 9.4 and 92.79% ± 4.07 of the vehicle control, respectively. However, in male rats, progesterone showed an impact on the capsaicin induced CGRP release. Specifically, in the dura mater tissue (Fig. 8C), progesterone trended towards augmenting the capsaicin induced release, with a CGRP level of 123.3% ± 11.4 of the vehicle control (*P* = 0.10). In contrast, in the TG tissue (Fig. 8D) progesterone significantly reduced the capsaicin induced release, down to 86.88% ± 4.19 of the vehicle control (*P* < 0.05).

In summary, while progesterone did not modify the capsaicin induced CGRP release in female rats, it exhibited a modulatory effect in male rats with a trend towards an increased release in the dura mater and a significant reduction in the TG.

### Ex vivo vasomotor responses of basilar arteries: myography

**Fig. 9:**
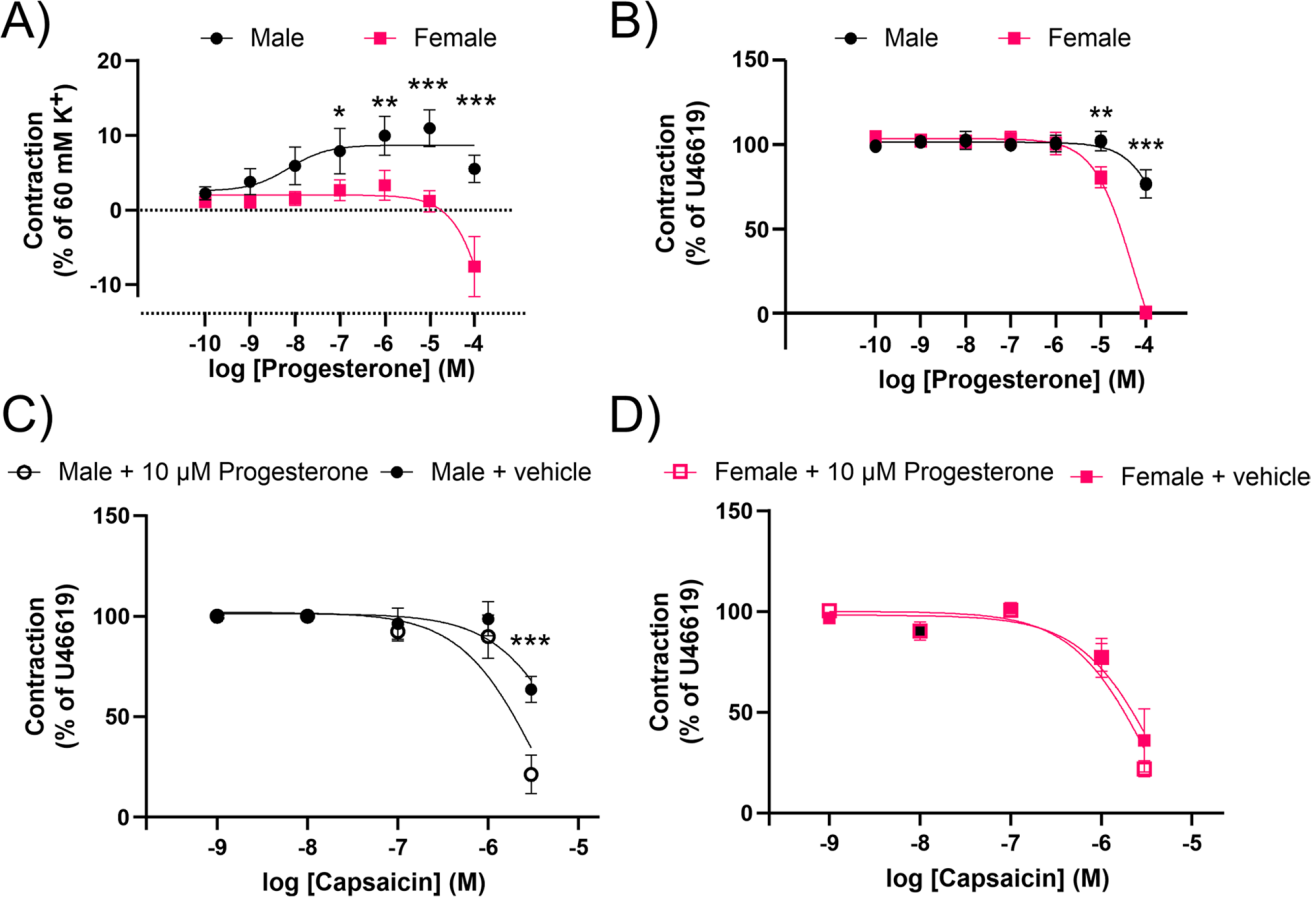
Progesterone modulation of capsaicin-induced vascular responses in rat basilar artery. **A** Depicts the contraction responses of male and female rats, expressed as a percentage of 60 mM K^+^. **B** Dilation responses following a pre-contraction with 100 nM U46619 are shown. Further, the differential capsaicin-induced responses in male (**C**) and female rats (**D**), following pre-treatment with 10 µM progesterone. **C** A significant increase in capsaicin-induced dilation is observed in male rats pre-treated with progesterone, while no significant difference was noted in female rats (**D**). Data are represented as mean ± SEM. A Two-way ANOVA with Sidak's post hoc test was used for statistical analysis

The effect of progesterone on male and female basilar artery segments was analyzed using a sensitive myograph system. It was found that progesterone elicited a weak contraction response in male arteries (Fig. 9A), with an average maximum contraction of 11.0 ± 2.4% at an EC_50_ concentration around 7.42 · 10^–9^ M (range: 1.03 · 10^–11^ to 6.59 · 10^–7^ M). Conversely, in female arteries (Fig. 9B), progesterone did not induce a contraction, but instead caused dilation at the highest concentrations. The apparent EC_50_ for this dilatory response was calculated to be 0.010 M. The maximum dilation observed was 7.6 ± 4.0%. These results suggest a sex-specific response to progesterone in the basilar artery segments, with contraction observed in males and dilation in females.

Further investigation of the potential modulatory effects of progesterone on capsaicin induced responses in rat basilar arteries was performed. Following an initial contraction induced by 100 nM of U46619, a concentration–response curve to capsaicin was established after application of either vehicle (DMSO) or 10 µM progesterone (in DMSO). In male basilar artery segments pre-treated with the vehicle, the residual contraction at the final concentration of 3 µM capsaicin was 63.5 ± 6.6% (Fig. 9C). However, when the segments were pre-treated with 10 µM of progesterone, a lower residual contraction of 21.3 ± 9.6% was observed at 3 µM capsaicin. This significant increase in dilation with progesterone pre-treatment (*P* < 0.001) suggests that progesterone could have a modulatory role enhancing capsaicin induced dilation in male rats.

Conversely, the female basilar artery segments (Fig. 9D) showed no significant difference in residual contraction at 3 µM capsaicin between vehicle (36.0 ± 15.7%) and progesterone (21.9 ± 4.1%) pre-treatment, indicating that progesterone did not modulate capsaicin induced dilation in female rats. Direct comparison between males and females did not reveal a significant difference in vasodilation induced by capsaicin; however, there was a numerical difference in the capsaicin induced dilation in the female basilar artery compared to males (*P* = 0.11, see Supplementary Fig. 2). These data thus highlight a sex-specific role of progesterone on capsaicin induced responses in rat basilar arteries, with an apparent enhancing effect on capsaicin induced dilation seen in males but not in females.

## Discussion

Presently, it is accepted that female sex hormones are involved in the pathophysiology of migraine, though the exact mechanism through with this occurs remain unclear [3, 33, 34]. However, estrogen withdrawal during the menstrual cycle has widely been accepted to play a role in migraine. It was reported that withdrawal in plasma estrogen levels during late luteal phase of the menstrual cycle induces migraine attacks in the women [35]. Additionally, there is currently no specific treatment to ameliorate menstrual-related migraine.

To the best of our knowledge this is the first study designed to analyze various aspects of the putative influence of progesterone and its progesterone receptor on different parts of the TVS. The most notable findings may open for discussions related to sex hormones in relation to primary headache disorders. We observed that only the PR-A isoform of progesterone receptors was expressed in the rat TG, as demonstrated by immunohistochemistry and RT-qPCR analysis. This is in agreement with a previous study by Mote and colleagues [36], which reported detection of PR-A by immunohistochemistry but failed to reveal PR-B in formalin fixed cell lines and tissues. This study reported that differences in protein folding might be responsible for masking the PR-B epitopes in formalin fixed tissues and therefore it could be the reason that the PR-A isoform only was detected by immunohistochemistry [36]. In addition, PR-A immunoreactivity was observed in most trigeminal neuron nuclei and cytoplasm, and in the cytoplasm of SGCs. Interestingly, female rat TGs had a significantly higher level of PR mRNA compared to the male equivalent. We observed that progesterone was prominently expressed in, or proximal to, the neuron cell membrane. Furthermore, a weaker expression was observed in the cytoplasm of trigeminal neurons and in affiliated Aδ-fibers. Since the observed expression was not uniform across all neurons, it is likely that progesterone can be either locally produced and released by this cell type, or that progesterone is unequally accumulated in certain cell types, which may be linked to the unequal expression of the PR-A receptor in the TG.

The effects of sex hormones in structures associated with migraine pain have often centered upon effects of estrogen. Here we demonstrate for the first-time co-expression of progesterone and its receptor (PR-A) with CGRP, RAMP1, and CLR in different parts of TVS. PR-A was mainly seen to co-express in RAMP1 and CLR-positive neurons, the two elements of the canonical CGRP receptor, but was not observed in Aδ -fibers (Fig. 5). Progesterone immunoreactivity was only weakly observed in relation to the small-medium sized neurons expressing CGRP. These results suggest that PR-A activation in the TG is focused on the neurons expressing the CGRP-receptor, which in turn could influence the onset, or progression, of migraine.

The close association of the different sex hormones and their relation to the menstruation cycle on one hand and the onset of menstruation as well as during pregnancy opens for interesting aspects on migraine pathophysiology [3]. During the menstrual cycle, the level of estradiol (the main female sex hormone) can vary by 200% relative to baseline. In comparison, the level of progesterone varies by over 1200% relative to lowest levels. The sex hormones estrogen, progesterone, and oxytocin drop just before the start of the menstruation, which may be a trigger for alterations in TVS associated with the onset of migraine attacks [3].

Progesterone itself was not found to induce CGRP release from dura mater or TG in male or female rats, indicating that this hormone does not directly trigger CGRP-dependent sensory neurotransmission. This aligns with previous research suggesting that progesterone primarily serves as a modulator rather than as an elicitor [37].

Little is known about the impacts of female sex hormones on the CGRP release in TVS [38]. Females has been shown to display notably higher basal CGRP release from the dura mater compared to males, indicating an inherent sex difference in CGRP release [39]. In a different study, no significant disparities in CGRP release between males and females were observed [6] but elevated baseline CGRP release in female rats during the pro-estrous state was noted, underscoring the potential influence of hormonal fluctuations in the female estrous cycle on CGRP levels. The current study aimed to investigate the effects of progesterone, and a paired design as percent of control was chosen, precluding parallel testing between males/females. However, a recent study observed a small but significant increase in CGRP release in the TGs of male and female rats, but no release was observed in the hemi-skulls when 1 µM of progesterone was applied [34]. In our study, we also observed no CGRP release when progesterone, in our case 10 µM, in the hemi-skulls. However, we did not observe any CGRP release in the TGs, which in contrast of the aforementioned study. These differences in results could be explained by the slight changes in setups, notably different use of buffers, vehicle, or controls. Further investigations are needed to conclude the effect of progesterone on CGRP release in the TVS. Nevertheless, one of the key findings in the present study was the tendency of enhanced capsaicin induced CGRP release observed in the dura mater preparation from male rats pre-treated with 10 µM progesterone. Similarly, in male basilar arteries, the addition of 10 µM progesterone significantly amplified the dilation in response to capsaicin. Capsaicin, a transient receptor potential vanilloid 1 (TRPV1) agonist, is known to induce the release of CGRP, leading to dilation of cerebral arteries [40]. The vasoconstrictor effect of capsaicin at the maximal concentration has been demonstrated in previous studies to show increases in Ca^2+^ [41]*.* This should have limited impact on capsaicin-induced vasodilation in basilar arteries due to the minimal additional constriction observed during pre-contraction, although the myograph measurements do not exclude the potential role of capsaicin-induced contraction as a contributing factor for the following results. The enhanced capsaicin response observed here suggests that progesterone may increase the sensitivity of these pathways, perhaps by modulating TRPV1 function. In contrast to these findings, an inhibitory effect of progesterone on capsaicin induced CGRP release was observed in male TG. This discrepancy, with the progesterone effects seen in the male dura mater and basilar arteries, warrants further investigation to unravel the underlying molecular mechanisms and potential tissue-specific roles of progesterone in these processes.

The myograph data showed a sex-specific difference in the EC_50_ value for progesterone effects in basilar artery dynamics. The male arteries demonstrated contraction at a much lower concentration of progesterone compared to the dilatory response observed in females at higher concentrations. This may reflect sex-specific differences in the expression or function of progesterone receptors or downstream signaling pathways in these tissues, a possibility that aligns with evidence of sex-based differences in steroid hormone effects on vascular function [6].

Little is known about potential effects of progesterone on TG neurons, and thus the cellular and subcellular localization of the sex hormones opens interesting aspects on gene regulation. Thus, progesterone has several physiological effects that are amplified in the presence of estrogens. Estrogens act through estrogen receptors (ERs) to induce or upregulate the expression of the PR [42]. One example of this is in breast tissue, where estrogens allow progesterone to mediate lobule-alveolar development [43, 44].

Elevated levels of progesterone potently reduce the sodium-retaining activity of aldosterone, resulting in natriuresis and reduction in extracellular fluid volume. Progesterone withdrawal, on the other hand, is associated with a temporary increase in sodium retention (reduced natriuresis, with an increase in extracellular fluid volume) due to the compensatory increase in aldosterone production, which combats the blockade of the mineralocorticoid receptor by the previously elevated level of progesterone [45].

Progesterone has been reported to be associated with anti-inflammatory, anti-vasoconstriction, and neuroprotective functions [45, 46]. Thus, this sex hormone may have widespread function in numerous parts of the body but here we have focused on the TVS and putative relation to primary headaches. Its close relation to the trigeminal system with location of its receptor to the Aδ-fibers and the CGRP containing neurons provide further elucidation of a modulatory role in headaches. The molecular details remain to be analyzed in detail.

Allopregnanolone (3α,5α-tetrahydro progesterone) is a major metabolite of progesterone that can be formed both de novo and from circulating precursors in neurons and glia [47]. Interestingly, allopregnanolone has been found to have a modulatory effect on synaptic and extra synaptic GABA_A_ receptors [48], which has been proposed to result in inhibition of neuronal excitability. Therefore, allopregnanolone has recently been approved for treatment of postpartum depression, which is postulated to occur from a steep drop in plasma progesterone after childbirth [49, 50]. Possibly, incubation with progesterone could lead to increased production of allopregnanolone, which in turn could account for the inhibitory effect on the capsaicin induced CGRP release that we observed in male TG.

## Conclusion

In conclusion, the present results demonstrate that the expression of progesterone and PR-A is more prominent in female TG as compared to the male. The results highlight the potential for progesterone to modulate sensory neurotransmission and vascular responses in a complex manner. These effects may vary by sex, tissue type, and the nature of the stimulus. Further investigations are needed to elucidate the underlying mechanisms and physiological implications of these findings.

### Supplementary Information

**Additional file 1: Supplementary Figure 1.**

**Additional file 2: Supplementary Figure 2.**

**Additional file 3: Supplementary Table 1.** Number of animals and tissues using in each experiment.

**Additional file 4: Supplementary Table 2.** CGRP release data in pg/ml.

## Data Availability

The datasets generated during and/or analyzed during the current study are available from the corresponding author on reasonable request.

## Abbreviations

BSA: Bovine serum albumin
CGRP: Calcitonin gene-related peptide
CNS: Central nervous system
cDNA: Complementary DNA
CLR: Calcitonin receptor-like receptor
DAPI: 4', 6-Diamidino-2-phenylindole
DNA: Deoxyribonucleic acid
DMSO: Dimethyl sulfoxide
ER: αestrogen receptor alpha
ER: βestrogen receptor beta
EIA: Enzyme Immunoassay
GPER: G-protein coupled estrogen receptor
GAPDH: Glyceraldehyde-3-phosphate dehydrogenase
GI Syn: Glutamine synthetase
mRNA: Messenger RNA
PBS: Phosphate buffer saline
PBS-T: Phosphate buffer saline-TritonX
PR: Progesterone receptor
PF: Paraformaldehyde
PVN: Paraventricular
TRPV1: Transient receptor potential vanilloid 1
RAMP1: Receptor activity-modifying protein 1
RNA: Ribonucleic acid
RT-qPCR: Real-time quantitative Polymerase Chain Reaction
SGC: Satellite glial cell
SON: Supraoptic nucleus
SIF: Synthetic interstitial fluid
TG: Trigeminal ganglion
TVS: Trigeminovascular system
TRPV1: Transient receptor potential vanilloid 1
