# Supplementary figures and images for: Progesterone distribution in the trigeminal system and its role to modulate sensory neurotransmission: influence of sex
Source: J Headache Pain. 2023 Nov 14;24(1):154. doi: 10.1186/s10194-023-01687-x (PMC10644471; doi:10.1186/s10194-023-01687-x)

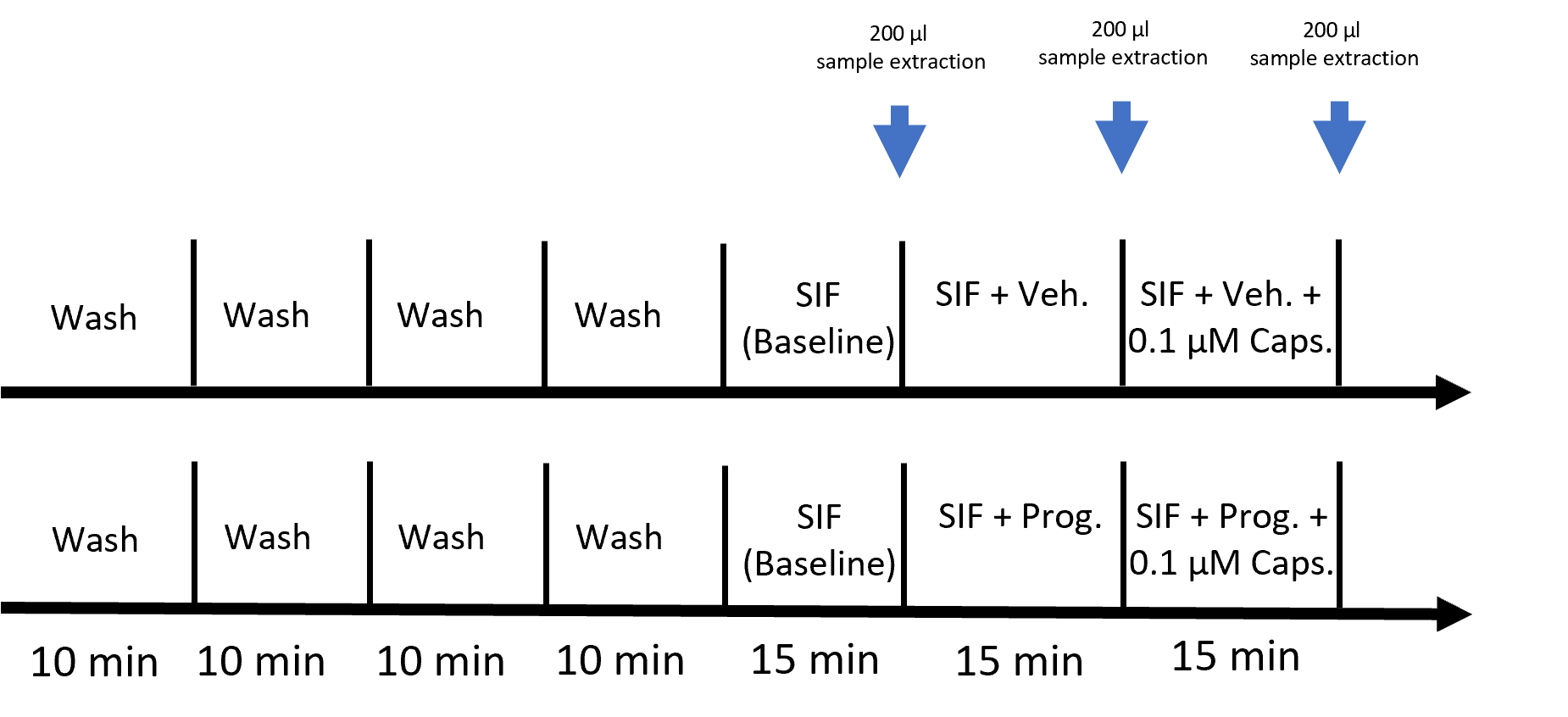

Supplement: Supplementary file 1 — Additional file 1: Supplementary Figure 1. [file 10194_2023_1687_MOESM1_ESM.tif]

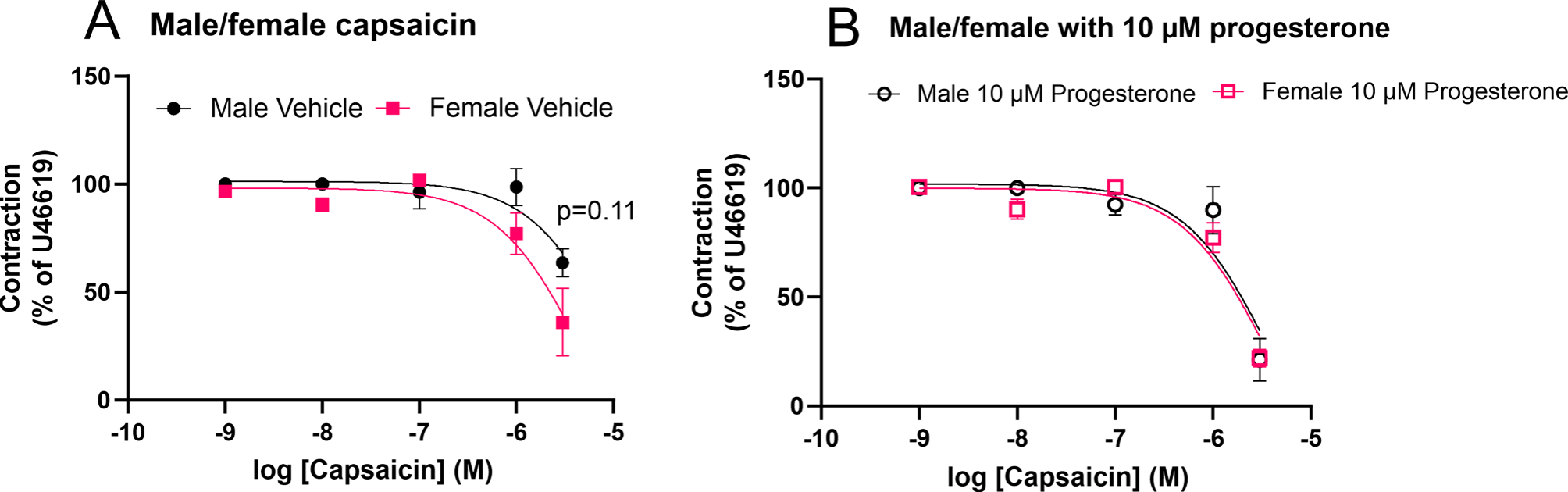

Supplement: Supplementary file 2 — Additional file 2: Supplementary Figure 2. [file 10194_2023_1687_MOESM2_ESM.tif]
