# Supplementary material for: Progesterone distribution in the trigeminal system and its role to modulate sensory neurotransmission: influence of sex
Source: J Headache Pain. 2023 Nov 14;24(1):154. doi: 10.1186/s10194-023-01687-x (PMC10644471; doi:10.1186/s10194-023-01687-x)
Supplement: Supplementary file 3 — Additional file 3: Supplementary Table 1. Number of animals and tissues using in each experiment. [file 10194_2023_1687_MOESM3_ESM.docx]

**Supplementary Table 1. Number of animals and tissues using in each experiment**

| **Experiments** | **Number of rats**  Male Female | **Tissues** |
| --- | --- | --- |
| Immunohistochemistry | 6 6 | TGs |
| RT-qPCR(1) | 8 - | TGs and SON(Hypothalamus) |
| RT-qPCR(2) | 3 3 | TGs |
| CGRP Release | 5 5 | TGs and dura mater |
| Myography | 12 12 | Basilar artery |
