# Supplementary material for: Progesterone distribution in the trigeminal system and its role to modulate sensory neurotransmission: influence of sex
Source: J Headache Pain. 2023 Nov 14;24(1):154. doi: 10.1186/s10194-023-01687-x (PMC10644471; doi:10.1186/s10194-023-01687-x)
Supplement: Supplementary file 4 — Additional file 4: Supplementary Table 2. CGRP release data in pg/ml. [file 10194_2023_1687_MOESM4_ESM.docx]

**Supplementary Table 2. CGRP release data in pg/ml**

|  | Vehicle baseline | Progesterone baseline | Vehicle | 10 µM Progesterone | Vehicle + 100 nM Capsaicin | 10 µM Progesterone  + 100 nM Capsaicin |  |  |  |
| --- | --- | --- | --- | --- | --- | --- | --- | --- | --- |
| Female TG: | 35.9 ± 5.4 | 31.4 ± 7.1 | 36.7 ± 1.7 | 39.5 ± 4.1 | 143.7 ± 20.2 | 142.7 ± 15.8 |  |  |  |
| Female Dura: | 27.8 ± 5.1 | 18.1 ± 7.5 | 29.0 ± 8.5 | 31.7 ± 4.4 | 45.1 ± 10.1 | 48.1 ± 5.6 |  |  |  |
| Male Dura: | 24.5 ± 2.1 | 12.9 ± 5.1 | 32.3 ± 4.5 | 31.2 ± 5.3 | 97.4 ± 3.6 | 120.9 ± 13.9 |  |  |  |
| Male TG: | 30.1 ± 6.4 | 28.9 ± 5.6 | 34.6 ± 5.6 | 39.4 ± 4.2 | 74.9 ± 7.6 | 65.0 ± 7.7 |  |  |  |

All data are for CGRP in pg/ml
